# Self-reporting of psychoneurophysical (PNP) symptoms in adults with four chronic diseases: a protocol for a scoping review

**DOI:** 10.1186/s13643-024-02498-0

**Published:** 2024-04-04

**Authors:** Carielle Joy Rio, Catherine Blumhorst, Catherine A. Kwiat, Christopher M. Nguyen, Alicia A. Livinski, Leorey N. Saligan

**Affiliations:** 1 Symptoms Biology Unit, Division of Intramural Research, National Institute of Nursing, Research, National Institutes of Health, Bethesda, MD USA; 2 National Institutes of Health Library, Office of Research Services, Office of the Director, National Institutes of Health, Bethesda, MD USA

**Keywords:** Chronic disease, Psychoneurophysical symptoms, Racial disparities, Self-reporting

## Abstract

**Background:**

Patient self-reporting of health-specific information, including symptoms, allows healthcare providers to provide more timely, personalized, and patient-centered care to meet their needs. It is critical to acknowledge that symptom reporting draws from the individual’s unique sociocultural background influencing how one perceives health and illness. This scoping review will explore whether racial groups with 4 chronic diseases (cardiovascular diseases, respiratory diseases, cancers, and diabetes) differ in self-reporting of psychoneurophysical (PNP) symptoms. The PNP symptoms of interest include depressive symptoms, fatigue, anxiety, pain, cognitive impairment, sleep impairment, mood impairment, irritability, and shortness of breath.

**Methods:**

Four databases will be searched by a biomedical librarian: CINAHL Plus (EBSCOhost), Embase (Elsevier), PubMed (NLM), Web of Science: Core Collection (Clarivate Analytics), and limited to publications written in the English language. Two independent reviewers will screen the records’ title, abstract, and then full text and extract the data from included articles using Covidence. A third reviewer will be used for resolving disagreements. Included articles must comprise adult patients with at least one of the specified chronic diseases who self-report at least one of the specified PNP symptoms. Studies that used clinician-administered questionnaires or obtained symptom responses from primary caregiver or patient designee will be excluded. Articles on patient-reported functionality or perceived quality of life will also be excluded from the review. Two reviewers will independently extract data (e.g., demographics, study design, racial group, chronic disease, measure/scale used for self-report) from each included article using Covidence and Microsoft Excel for data cleaning and analyses.

**Discussion:**

This scoping review may potentially identify the relevant and practical implications related to clinical decision-making and health outcomes for patients experiencing the psychoneurophysical symptoms included in this study. The authors will present how the results can be utilized in clinical practice, health policy, and research planning.

**Systematic review registration:**

The protocol was registered on Open Science Framework (OSF) at: https://osf.io/ps7aw

## Background

The high prevalence of individuals with chronic diseases globally is creating significant health and economic concerns and pressures in countries around the world [1]. Seven of the 10 leading causes of death identified by the World Health Organization (WHO) in 2019 are non-communicable or chronic diseases including ischemic heart disease, stroke, chronic obstructive pulmonary disease, cancers, Alzheimer’s disease, diabetes mellitus, and kidney disease [2]. In 2022, the WHO reported that while life expectancy has remained constant since 2000, the number of deaths due to chronic diseases has increased from 60.8% in 2000 to 73.6% in 2019 [2]. In 2019, cardiovascular diseases, cancers, chronic respiratory diseases, and diabetes resulted in approximately 33.2 million deaths [2]. The combined income loss of workers in the United States (US) who experience functional limitations secondary to chronic diseases is estimated at 4.95 billion US dollars [3]. The national annual spending of the US for chronic diseases, along with mental health conditions, amounts to 4.1 trillion US dollars or 90% of the yearly US healthcare expenditure [4]. More than 300 billion US dollars was spent on cardiovascular diseases alone between 2017 to 2018 [5]. These data show that chronic diseases present serious health and economic consequences.

The rising number of people living with chronic diseases prompted a shift from appointment-based consultations to patient-driven models of care where patients actively participate in monitoring and managing their own medical conditions [6]. In recent years, there has been increasing use of technologies primarily intended to facilitate symptom reporting by patients to their healthcare providers for faster clinical decision-making [6, 7]. Patient self-reporting of health-specific information, including symptoms, allows healthcare providers to provide more timely, personalized, and patient-centered care that focuses on the patient’s physical, emotional, and practical needs. Further, patient-reporting of health information also facilitates respect and consideration of the patient’s values and preferences in planning for their care [8].

Addressing chronic diseases requires accounting for social determinants of health that influence health outcomes. Disparities in estimated chronic disease prevalence are linked to disparities involving socioeconomic, racial, and ethnic groups [9]. Poor health outcomes (i.e., controls of hypertension, hyperglycemia, hyperlipidemia, and HIV) are found to be more prevalent in minority populations [10]. The disparity in chronic disease control between races is attributed to differences in health literacy, beliefs regarding medical treatment, perceived discrimination, and access to high-performing insurance plans [10]. It is critical to acknowledge that symptom reporting draws from the individual’s unique sociocultural background influencing how one perceives health and illness. It has been established that variations in symptom experience, including interpretation and reporting, exist across cultures and contexts [11].

A study in England found that Romanians and Southeast Asians suppress symptoms of anxiety and depression due to the cultural stigma attached to these conditions, but instead report physical pain symptoms that are challenging to address [12]. These racial and cultural variations have been documented not only in mental health care assessment but also in other health conditions such as reporting menopausal symptoms, and cancer treatment experience [13–15]. Earlier studies found that race influences patients’ perception of symptom intensity, as well as the burden from the symptoms being experienced [6]. Low proficiency in the predominant language used in the healthcare setting, which is a significant limiting factor for immigrant populations, remains a major challenge in patient-reported approaches [7]. Understanding health behaviors such as symptom reporting ought to consider race, not only based on genetic inheritance, but also consider the disparity in the socioeconomic factors that may intersect with race [16]. Lack of access to insurance, fear of discrimination, language barrier, low literacy levels, and cultural acceptability of certain medical interventions are among the factors driving racial disparities in symptom reporting [7, 10, 12].

## Aim and review question

Even with the strong evidence of racial disparities in patient reporting of symptoms, there is a scarcity of studies that synthesize earlier studies that explored this phenomenon. This information is critical to propose changes not only to improve our clinical practice but also to upgrade our educational milieu and revamp our research perspectives. Therefore, the aim of this review is to explore whether racial groups with four chronic diseases differ in self-reporting psychoneurophysical (PNP) symptoms. This scoping review will focus on the primary question “Do racial groups with 4 chronic diseases differ in self-reporting PNP symptoms?”.

## Methods

### Eligibility criteria

The following chronic diseases will be included (1) cardiovascular diseases such as ischemic heart disease or coronary artery disease, chronic heart failure, hypertension, chronic arrhythmias, peripheral vascular disease, aneurysms, and stroke; (2) respiratory diseases including chronic obstructive pulmonary diseases, interstitial pulmonary fibrosis, and asthma; (3) all cancers, and; (4) diabetes mellitus types 1 and 2. We selected these four chronic diseases as they are the leading causes of chronic disease-related deaths worldwide according to the WHO. The PNP symptoms we will include are depressive symptoms, chronic fatigue (e.g., physical, cognitive, mental), anxiety (e.g., panic disorder, general anxiety disorder, social anxiety disorder, specific phobias, separation anxiety disorder), chronic pain, cognitive impairment (e.g., mild cognitive impairment, subjective cognitive decline, brain fog, chemo brain, dementia), sleep impairment (e.g., insomnia, poor sleep quality, early waking, circadian rhythm disorders, parasomnias, sleep-related movement disorders, sleep-related breathing disorders), mood impairment (e.g. negative affect, tension, anger, emotional lability) irritability, and shortness of breath.

The review will only include articles on original research. Quantitative, qualitative, and mixed-method studies will be considered for inclusion. Editorials and commentaries will be excluded. Review articles will also not be included; however, the reference list of review articles will be scanned for original research articles that will be included for title, abstract, and full-text screening. A search for grey literature will also be conducted. This step may include full-text conference proceedings, preprints, dissertations, government documents/reports, and technical reports. Only articles published in English will be included as the review team does not have translation capabilities available.

### Search strategy

The search strategy will aim to locate published primary studies and systematic reviews. An initial limited search of PubMed (US National Library of Medicine) was undertaken to identify articles on the topic to assist with keyword selection. The text words contained in the titles, abstracts, full text, and the controlled vocabulary terms used to describe the relevant articles were used to develop a full search strategy.

The databases CINAHL Plus (EBSCOhost), Embase (Elsevier), PubMed (US National Library of Medicine), and Web of Science: Core Collection (Clarivate Analytics) will be searched by a biomedical librarian. The search strategy, including all identified keywords and controlled vocabulary terms (CINAHL Subject Headings, EMTREE, MeSH), will be adapted for each included information source (see Table 1 for the final search strategy used for PubMed).

**Table 1:** Search strategy

	Concept	Search strategy	Totals
#1	Patient report	(“self report*”[tiab] OR “self-report*”[tiab] OR Self Report[Mesh] OR “patient report*”[tiab] OR “patient-report*”[tiab] OR "Patient Reported Outcome Measures"[Mesh])	284,423
#2	Symptom	(symptom*[tiab] OR "Signs and Symptoms"[Mesh:NoExp])	1,463,218
#3	PNP	(psychoneurophys*[tiab] OR depression[tiab] OR depressive[tiab] OR “chronic fatigue”[tiab] OR anxiety[tiab] OR “panic disorder*”[tiab] OR “panic attack*”[tiab] OR phobia*[tiab] OR “phobic disorder*”[tiab] OR “chronic pain*”[tiab] OR “cognitive impair*”[tiab] OR “cognitive dysfunction*”[tiab] OR “cognitive decline*”[tiab] OR “brain fog*”[tiab] OR “chemo brain”[tiab] OR “mental fatigue”[tiab] OR dementia*[tiab] OR “sleep impairment*”[tiab] OR Insomnia[tiab] OR insomniac*[tiab] OR “poor sleep”[tiab] OR “sleep wake disorder*”[tiab] OR “sleep disorder*”[tiab] OR parasomnia*[tiab] OR “early waking”[tiab] OR “early awakening”[tiab] OR sleeplessness[tiab] OR dyssomnia*[tiab] OR hypersomnia*[tiab] OR “circadian rhythm disorder*”[tiab] OR parasomnia*[tiab] OR “sleep-related movement disorder*”[tiab] OR “sleep related movement disorder*”[tiab] OR “sleep movement disorder*”[tiab] OR “sleep-related rhythmic movement disorder*”[tiab] OR “restless leg*”[tiab] OR “sleep related breathing disorder*”[tiab] OR “sleep-related breathing disorder*”[tiab] OR “sleep apnea*”[tiab] OR “sleep disordered breathing”[tiab] OR “mood impairment*”[tiab] OR “psychotic affective disorder*”[tiab] OR “mood disorder*”[tiab] OR “negative affect*”[tiab] OR “poor self-concept”[tiab] OR “emotional distress*”[tiab] OR tension[tiab] OR tense[tiab] OR anger[tiab] OR angry[tiab] OR “emotional labilit*”[tiab] OR “rapid mood change*”[tiab] OR irritabilit*[tiab] OR irritable[tiab] OR “shortness of breath”[tiab] OR dyspnea*[tiab] OR "Irritable Mood"[Majr] OR "Dyspnea"[Majr] OR "Anger"[Majr] OR "Affective Disorders, Psychotic"[Majr] OR "Sleep Apnea Syndromes"[Majr] OR "Restless Legs Syndrome"[Majr] OR "Sleep Initiation and Maintenance Disorders"[Majr] OR "Sleep Wake Disorders"[Majr] OR "Parasomnias"[Majr] OR Dyssomnias[Majr] OR "Sleep Disorders, Intrinsic"[Majr] OR "Dementia"[Majr] OR "Mental Fatigue"[Majr:NoExp] OR "Cognitive Dysfunction"[Majr] OR "Phobic Disorders"[Majr] OR "Panic Disorder"[Majr] OR "Fatigue Syndrome, Chronic"[Majr] OR "Anxiety"[Majr] OR "Anxiety Disorders"[Majr:NoExp] OR "Depression"[Majr] OR "Depressive Disorder"[Majr])	1,355,318
#4	Chronic disease	(“chronic disease*”[tiab] OR “cardiovascular disease*”[tiab] OR “Ischemic heart disease*”[tiab] OR “heart attack*”[tiab] OR “myocardial infarction*”[tiab] OR angina[tiab] OR anginas[tiab] OR “coronary artery disease*”[tiab] OR “chronic heart failure”[tiab] OR arrythmia*[tiab] OR “peripheral vascular disease*”[tiab] OR aneurysm*[tiab] OR stroke[tiab] OR strokes[tiab] OR “cerebrovascular accident*”[tiab] OR “brain vascular accident*”[tiab] OR “brain infarct*”[tiab] OR “respiratory disease*”[tiab] OR COPD[tiab] OR “chronic obstructive pulmonary disease*”[tiab] OR “chronic obstructive airway disease*”[tiab] OR “chronic airflow obstruction*”[tiab] OR “chronic bronchitis”[tiab] OR emphysema*[tiab] OR “interstitial pulmonary fibros*”[tiab] OR asthma[tiab] OR asthmas[tiab] OR cancer[tiab] OR cancers[tiab] OR neoplasm*[tiab] OR malignan*[tiab] OR neoplasia*[tiab] OR diabetes[tiab] OR "Cardiovascular Diseases"[Majr:NoExp] OR "Heart Diseases"[Majr:NoExp] OR "Arrhythmias, Cardiac"[Majr:NoExp] OR "Heart Failure"[Majr:NoExp] OR "Myocardial Ischemia"[Majr:NoExp] OR "Angina Pectoris"[Majr:NoExp] OR "Coronary Disease"[Majr:NoExp] OR "Myocardial Infarction"[Majr:NoExp] OR "Peripheral Vascular Diseases"[Majr:NoExp] OR "Aneurysm"[Majr:NoExp] OR "Stroke"[Majr:NoExp] OR "Asthma"[Majr:NoExp] OR "Bronchitis, Chronic"[Majr:NoExp] OR "Pulmonary Disease, Chronic Obstructive"[Majr:NoExp] OR "Emphysema"[Majr:NoExp] OR "Pulmonary Emphysema"[Majr:NoExp] OR "Pulmonary Fibrosis"[Majr:NoExp] OR "Neoplasms"[Majr:NoExp] OR "Diabetes Mellitus"[Majr:NoExp] OR "Diabetes Mellitus, Type 1"[Majr] OR "Diabetes Mellitus, Type 2"[Majr])	5,231,424
#5		#1 AND #2 AND #3 AND #4	4948
#6		#5 AND English[lang]	4912
#7		#6 NOT (letter[ptyp] OR editorial[ptyp] OR comment[ptyp] OR news[ptyp] OR editorial[tiab] OR commentary[tiab] OR "Published Erratum"[Publication Type] OR errata[tiab] OR erratum[tiab] OR corrigenda[tiab] OR corrigendum[tiab] OR protocol[ti] OR protocols[ti] OR “meta-analysis”[tiab] OR “meta-analyses”[tiab] OR metanalyses[tiab] OR metanalysis[tiab] OR metaanalyses[tiab] OR metaanalysis[tiab] OR “meta analyses”[tiab] OR “meta analysis”[tiab] OR "Review"[Publication Type] OR “systematic review*”[tiab] OR "Systematic Review"[Publication Type] OR "Meta-Analysis" [Publication Type] OR "Network Meta-Analysis"[Mesh] OR “integrative review”[tiab])	4506

Search strategy for PubMed (US National Library of Medicine)

Date of search: December 19, 2023

Total retrieved with limits = 4506

Additionally, the reference lists of articles included in the review and relevant reviews will be scanned for any additional articles, and will proceed through the screening process.

### Study selection

EndNote 20 (Clarivate Analytics) will be used by the biomedical librarian to collect, organize, remove duplicates, and identify unique records for screening. Covidence (Veritas Health Innovations) will be used for the study screening steps and data extraction. Microsoft Excel will be used for data cleaning and analyses. A pilot test of the screening step will be conducted in Covidence with all reviewers using a random sample of 20 records selected by the biomedical librarian. Screening will occur in two stages: (1) title and abstracts and (2) full text. After the pilot test, the team will meet and review the process, address questions, and update the eligibility criteria as necessary.

Once the questions during the pilot test are addressed and the steps are finalized, the titles and abstracts of all records will be screened by two reviewers independently using the inclusion criteria. For the full-text review, the full text of those articles included after the title and abstract screening will be obtained and uploaded into Covidence. Next, two reviewers will independently screen the full text of all records using the eligibility criteria. Reasons for exclusion of full-text papers that do not meet the inclusion criteria will be recorded and reported in the PRISMA flow diagram. All conflicts at each stage will be resolved by a third reviewer or by the team, where consensus will be obtained by the majority of the team members present.

### Data extraction

To pilot the data collection process, all team members will independently extract data from the same five articles selected by the biomedical librarian from the included articles. After extracting the data, the team will meet to assess whether the data items can be extracted correctly, whether any relevant data items are missing, ease of use, and challenges encountered while using the tool and revise the data extraction process, as necessary. If changes are necessary, the data collection form may be revised and re-tested using another set of five randomly selected articles. Any modifications made to the data extraction tool and process will be fully documented in the protocol and eventual manuscript. Once the data collection form is finalized, the authors will proceed to extract the data from all included articles (Table 2).

**Table 2:** Data extraction tool

First Author Last Name	Publication Year	Study Design	Study Location (state or country)	Total Study Sample Size (n)	Sample Size of each Racial/Ethnic Group (n) in Study [use US OMB categories]	Chronic disease/s (4 specific: Cardiovascular, Respiratory, Cancer, Diabetes)	PNP symptoms (specified symptoms)	Variables and Measure/Scale used for Patient Self-Reporting	Significant findings	Comments

Data from the included articles will be extracted by two reviewers independently using a data extraction tool developed by the review team in Covidence. Any discrepancies or conflicts between the reviewers will be resolved by a third reviewer or by discussion with the team. Data that is missing or unclear will be marked as either not reported or not available.

### Data analysis

The data items to collect from each included article are first author’s last name; publication year; study design; study location; total study sample size; sample size for each racial/ethnic group; specific chronic disease studied; PNP symptoms reported; variables and measure/scale used for self-reporting; and general comments. In this review, race will be determined based on origin and/or self-identification, regardless of the person’s geographic location. Categories of race will be based on the National Institutes of Health (NIH) general guidelines on Race and National Origin [17]. We will not assess the risk of bias of the individual studies included in this review as this step is optional when conducting a scoping review.

### Presentation

We will attempt to characterize how and which PNP symptoms are self-reported, for which chronic diseases, and if there are reported differences in self-reporting of the PNP symptoms across racial groups. Tables will be utilized to present the key summary characteristics of the included studies, as well as their major findings that address the research aims. A narrative summary to accompany tables and figures will describe the relation of the results to the scoping review’s research question and aims. Figures may be used to provide a graphical presentation of the different themes formulated based on the analysis.

### Protocol and registration

The proposed scoping review will be conducted in accordance with the JBI methodology for scoping reviews [18] and reported using the Preferred Reporting Items for Systematic Reviews and Meta-Analyses extension for Scoping Reviews (PRISMA-ScR) [19]. A protocol was written a priori using the PRISMA Protocol template and was registered on Open Science Framework (https://osf.io/ps7aw). The scoping review team will include a biomedical librarian as a methodological expert, a nurse symptom scientist as the content expert, a nurse practitioner, and a doctorally-prepared nurse who will work in tandem with two post-baccalaureate fellows, as reviewers. The team will meet weekly during the screening and data collection steps. Additional experts may be invited during the consultation and dissemination stages of this review.

## Discussion

We hope to identify the relevant and practical implications of the scoping review results as related to clinical decision-making and health outcomes. The authors will present how the results can be utilized in clinical practice, health policy, and research planning. Recommendations on future studies may be formulated, not only based on the results related to the research question but also based on the gaps in present evidence identified during the review.

## Data Availability

Data sharing is not applicable to this article as it is a protocol for a scoping review and no datasets were generated or analyzed.

## Abbreviations

JBI: Joanna Briggs Institute
MeSH: Medical Subject Headings
PNP: Psychoneurophysical
PRISMA: Preferred Reporting Items for Systematic Reviews and Meta-Analyses
PRISMA-ScR: Preferred Reporting Items for Systematic reviews and Meta-Analyses extension for Scoping Reviews
US: United States of America
WHO: World Health Organization
